# Complete Genome Sequence of Human Oral *Actinomyces* sp. HMT175 Strain ORNL0102, a Host of the Saccharibacterium (TM7) HMT957

**DOI:** 10.1128/MRA.00412-21

**Published:** 2021-06-10

**Authors:** Snehal Joshi, Peter T. Podar, Floyd E. Dewhirst, Mircea Podar

**Affiliations:** a Biosciences Division, Oak Ridge National Laboratory, Oak Ridge, Tennessee, USA; b Department of Microbiology, University of Washington, Seattle, Washington, USA; c School of Engineering, Vanderbilt University, Nashville, Tennessee, USA; d Department of Microbiology, The Forsyth Institute, Cambridge, Massachusetts, USA; e Department of Oral Medicine, Infection, and Immunity, Harvard School of Dental Medicine, Boston, Massachusetts, USA; Queens College CUNY

## Abstract

*Actinomyces* sp. HMT175 strain ORNL0102 was isolated from a human saliva sample and can serve as a host for the ectobiont saccharibacterium (TM7) HMT957. Its 3.3-Mbp circular chromosome was completely sequenced using PacBio long reads, and it encodes 2,408 proteins and 63 RNAs.

## ANNOUNCEMENT

At least two dozen *Actinomyces* species (phylum *Actinobacteria*) are known to colonize the human body, primarily the oral cavity. Some strains have not been formally described, and some oral *Actinomyces* strains are still refractory to isolation in pure culture (1, 2). *Actinomyces* strains can cause specific infections (actinomycoses), and some have also been linked to oral diseases (gingivitis, periodontitis, and caries) (1, 3–5). Oral *Actinomyces* strains can serve as hosts for epibiotic/parasitic saccharibacteria (TM7), and there is some degree of specificity in those interactions (6–10). *Actinomyces* sp. HMT175 strain ORNL0102 was coisolated with the saccharibacterium (TM7) HMT957 as part of a project for high-throughput cultivation of human oral bacteria (10) and may represent a yet-undescribed species. Here, we report its complete genome sequence. Human subject recruitment and sampling protocols were approved by the Oak Ridge Site-Wide and Forsyth Institute institutional review boards. Written, informed consent was obtained from all participants.

*Actinomyces* sp. HMT175 strain ORNL0102 was grown anaerobically (90% N_2_/10% CO_2_) in 100 ml brain heart infusion (BHI) medium (Difco) for 2 days at 37°C. Genomic DNA was isolated using a proteinase K-SDS digestion and phenol-chloroform extraction protocol (11). DNA sizing using a Femto Pulse system (Agilent) revealed a broad peak (15 to 25 kb) with fragments extending to >90 kb. The genomic library was prepared without size selection, using the SMRTbell template preparation kit v1.0 (Pacific Biosciences [PacBio], Menlo Park, CA), and sequenced on a PacBio Sequel instrument using a Sequel II 8M single-molecule real-time (SMRT) cell (30-h run). Default parameters were used for all software unless otherwise specified. The sequence reads were filtered based on quality values and assembled using HGAP4 in PacBio SMRTLink v7. A total of 88,272 polymerase reads (mean length, 54,332 nucleotides) and 462,086 subreads (*N*_50_, 9,207 nucleotides) were used in the assembly, generating a single 3-Mbp contig, with a mean confidence value (quality value) of 88.3 and mean coverage of 1,449-fold. To determine whether the chromosome is circular, we synthesized a pair of oligonucleotides (175F, 5′-GTTCATCAGACCGCTTCAAG; 175R, 5′-CGGTTGTAGTTGACGGTCTG) to amplify outward from the 5′ and 3′ ends of the contig, and we sequenced the resulting 0.8-kbp PCR product using Sanger chemistry (Eurofin Genomics LLC). Mapping of the reads using Geneious Prime 2020 (12) identified the continuity of the original contig ends and confirmed that the chromosome is circular and 3,024,208 bp long, with a G+C content of 68.9%.

**FIG 1:**
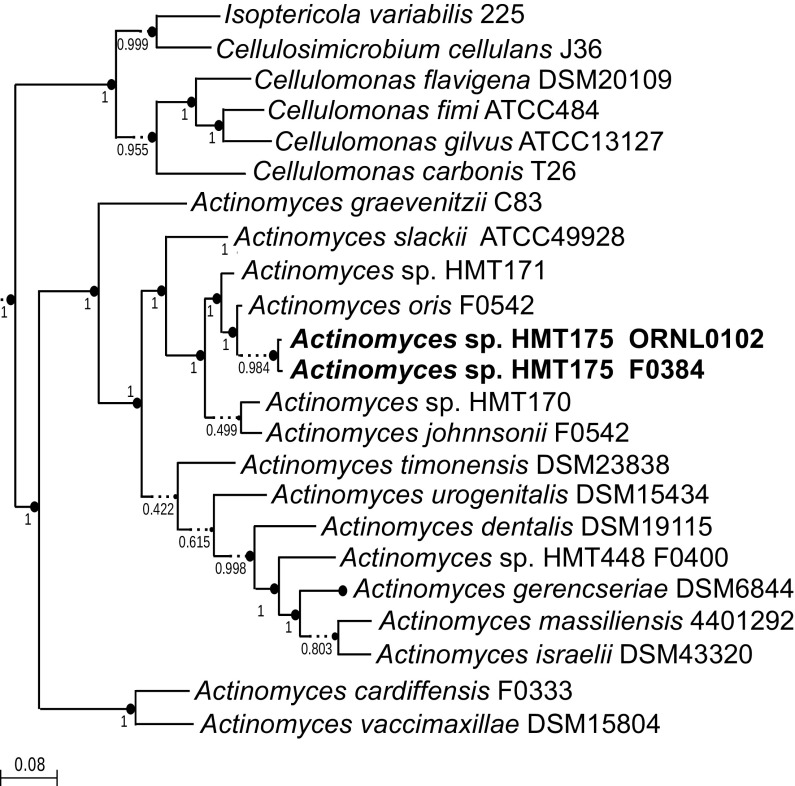
Phylogenetic tree of *Actinomyces* sp. HMT175 strain ORNL0102 and related human oral *Actinomyces* strains based on 49 core, universal bacterial proteins, using FastTree2 in KBase. Numbers at the nodes indicate support values.

To predict and to annotate the genes, we used the NCBI Prokaryotic Genome Annotation Pipeline (PGAP) v5.0 (13). The chromosome encodes 2,408 proteins, 51 tRNAs, 3 rRNA operons, and 6 other predicted small and regulatory RNAs. The genome was compared with other human oral *Actinomyces* genomes using FastANI v0.1.2 and SpeciesTreeBuilder v1.0 implemented in KBase (14). Based on the average nucleotide identity (ANI) of 96.7% (15) between strain ORNL0102 and the draft genome of another HMT175 strain isolated at the Forsyth Institute (F0384 [GenBank accession number NZ_AFUR00000000.1]), both isolates may belong to an undescribed *Actinomyces* species most closely related to Actinomyces oris F0542 (Fig. 1), with an ANI of 95%. The genome data will help to study the diversification of oral *Actinomyces* strains and the specificity of interactions between *Actinomyces* and saccharibacterial/TM7 species.

### Data availability.

The *Actinomyces* sp. HMT175 strain ORNL0102 genome sequence has been deposited in GenBank under the accession number CP068012. The version described in this paper is the first version, CP068012.1. The PacBio reads have been deposited in SRA under the accession number SRX10635573.
